# Echocardiographic Contrast Delineation of Left and Right Ventricular Dissection: Insights on Broken Boundaries

**DOI:** 10.1016/j.jaccas.2025.106720

**Published:** 2026-01-17

**Authors:** Cole Block, Nikhil Gandhi, Gerald Cohen

**Affiliations:** Department of Cardiology, Henry Ford St John Hospital, Detroit, Michigan, USA

**Keywords:** contrast echocardiography, left ventricular septal dissection, myocardial infarction, right ventricular dissection, ventricular septal defect

## Abstract

**Background:**

Post–myocardial infarction (MI) ventricular septal defect (VSD) is a life-threatening complication that may involve ventricular septal dissection. There are few reports describing combined VSD and right ventricular free wall dissection or the use of echocardiographic contrast to delineate this pathology.

**Case Summary:**

A 77-year-old patient with a history of coronary artery bypass presented with MI. Two-dimensional echocardiography and echocardiographic contrast-enhanced imaging helped to delineate a VSD with a complex dissection network extending into the right ventricular free wall.

**Discussion:**

This case underscores the importance of echocardiographic contrast agents for prompt and thorough evaluation of post-MI dissection networks, which was critical for facilitating diagnosis and guiding clinical decision-making.

**Take-Home Message:**

This case highlights the use of echocardiographic contrast agents to delineate a complex dissection network involving both ventricles originating from a VSD in a patient after MI.

## Visual Summary

**Figure:**
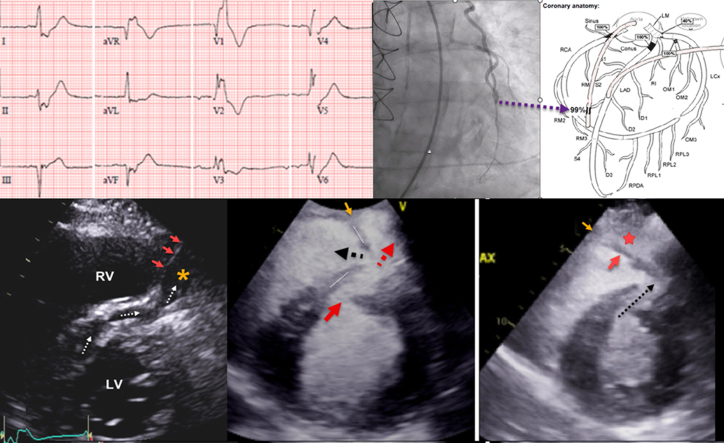

Post–myocardial infarction (MI) ventricular septal defect (VSD) is a rare but life-threatening complication that may involve ventricular septal dissection. Prompt identification and evaluation is critical because early surgical or percutaneous intravascular intervention may reduce mortality.

Transthoracic echocardiography (TTE) with Doppler remains the initial imaging modality of choice given its cost-effectiveness, versatility, and high diagnostic accuracy. A previous case report^1^ described the combined use of two-dimensional and contrast-enhanced echocardiography for the evaluation of ischemic VSD in a patient with poor echocardiographic windows and a simple through-and-through VSD. Our case demonstrates a unique example of complex post-MI VSD with concomitant left ventricular (LV) and anterior right ventricular (RV) free wall dissection.

**Visual Summary:**
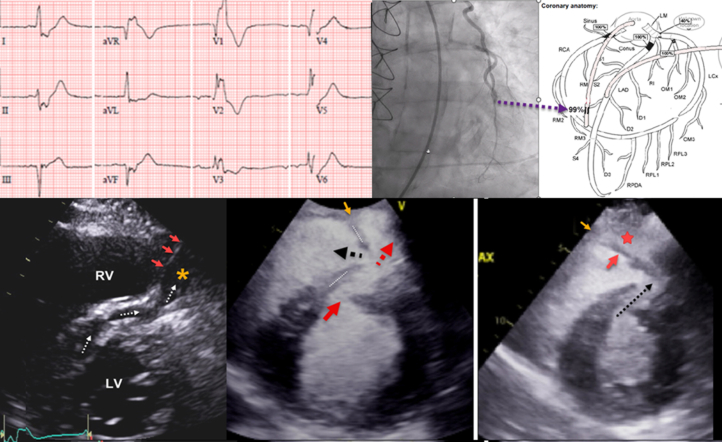
A 77-Year-Old Patient With a History of Coronary Artery Bypass Presented With Myocardial Infarction Two-dimensional echocardiography and echocardiographic contrast-enhanced imaging helped to delineate a ventricular septal defect, with a complex dissection network extending into the right ventricular free wall. LV = left ventricle; RV = right ventricle.

## History of Presentation

A 77-year-old man with a history of hypertension, hyperlipidemia, and remote coronary artery bypass grafting presented with 2 days of substernal chest pain that was exacerbated with physical activity. On arrival, he had a blood pressure of 86/53 mm Hg, a heart rate of 99 beats/min, a respiratory rate of 20 breaths/min, and an oxygen saturation of 100% on room air. A grade 3 systolic ejection murmur without rubs or gallops was present, and lungs were clear bilaterally.

**Take-Home Message**

• This case highlights the use of echocardiographic contrast agents to delineate a complex dissection network involving both ventricles originating from a ventricular septal defect in a post–myocardial infarction patient.

## Past Medical History

The patient was a nonsmoker with a history of hypertension, hyperlipidemia, and remote coronary artery bypass grafting.

## Differential Diagnosis

Given the patient's prior history of obstructive coronary artery disease and presenting symptoms, acute coronary syndrome was considered the most likely diagnosis. The differential included aortic dissection, pericarditis, musculoskeletal pain, and pulmonary embolism.

## Investigations

**Figure 1:**
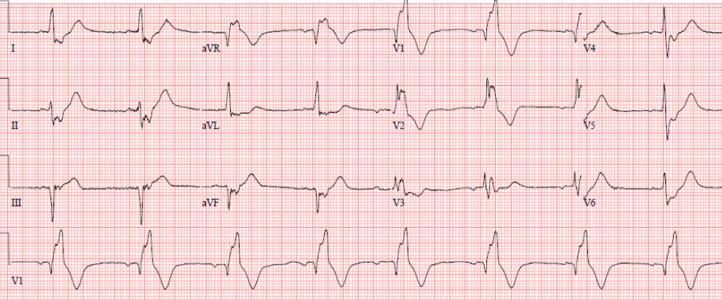
12-Lead Electrocardiogram in the Emergency Department Before Coronary Catheterization ECG demonstrates a low atrial or junctional rhythm at 52 beats/min, with left anterior fascicular block and right bundle branch block, ST-segment elevation in leads AVR and V_2_, and ST-segment depression in leads II and AVF. ECG = electrocardiogram.

Electrocardiogram (Figure 1) showed an ectopic atrial rhythm with left anterior fascicular block and right bundle branch block, and ST-segment elevation in leads AVR and V_2_. Initial troponin-T level was elevated at 2.92 ng/mL. The patient received 325 mg of aspirin and a 4,000-unit heparin bolus in the emergency department. He was urgently transported for cardiac catheterization before emergent echocardiography could be performed.

**Figure 2:**
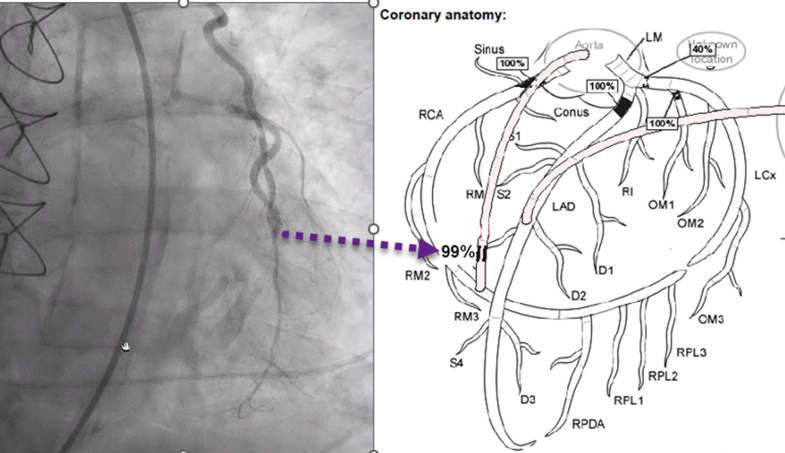
Occlusion During Left Heart Catheterization Left heart catheterization showed 99% occlusion of the SVG to the distal RCA at the distal anastomosis, with TIMI flow grade 1 and no visualization of the more distal vessel. RCA = right coronary artery; SVG = saphenous vein graft.

Emergent coronary angiography revealed acute occlusion of a saphenous vein graft to the right coronary artery and a patent left internal mammary artery graft to the left anterior descending artery. Balloon angioplasty of the right coronary artery graft was performed without stenting owing to severe distal vessel disease (Figure 2). Right heart catheterization demonstrated an oxygen step-up from the right atrium (30%) to the pulmonary artery (77%), indicating a left-to-right shunt. An intra-aortic balloon pump was placed to support coronary perfusion and LV function.

**Figure 3:**
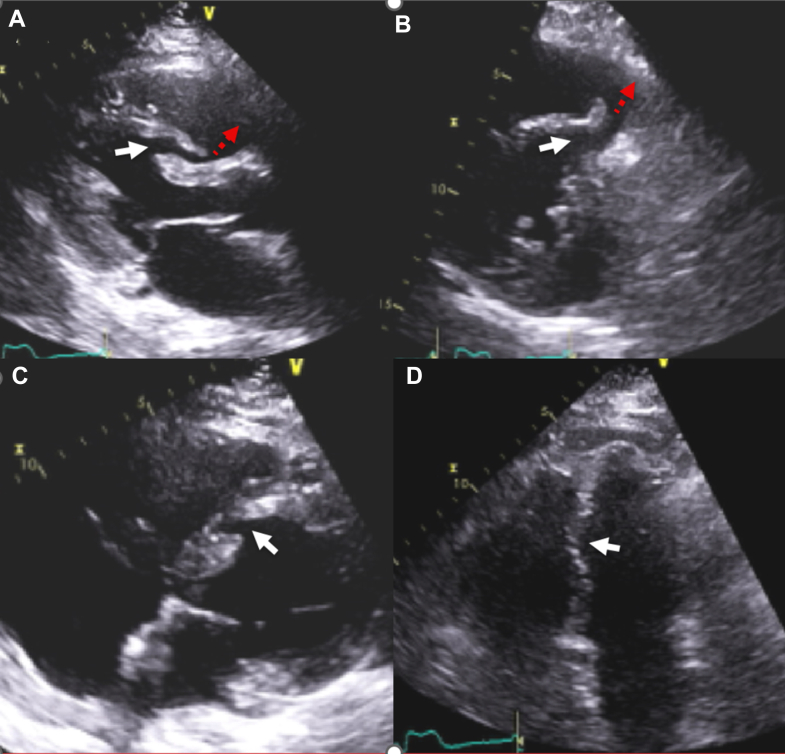
Postintervention TTE Showing Mid Cavity VSD (A and B) Parasternal long-axis and short-axis views show midcavity VSD (white arrow) dissection through the septum toward the RV (red arrows). (C and D) Four-chamber views show midcavity VSD inlet (white arrows). RV = right ventricle; TTE = transthoracic echocardiography; VSD = ventricular septal defect.

**Figure 4:**
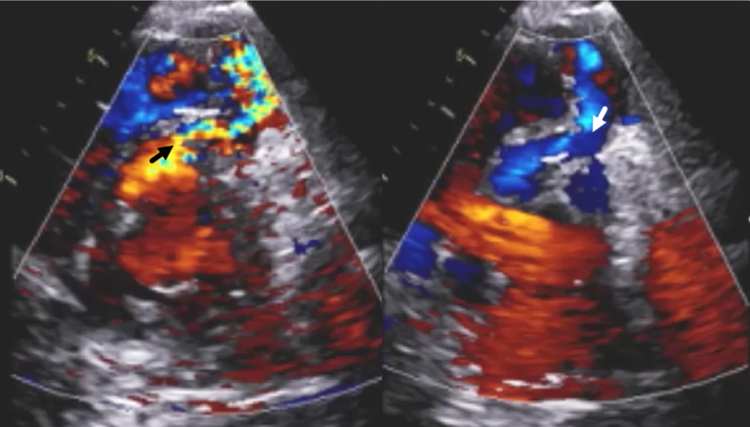
Postintervention TTE Showing To-and-Fro Flow Parasternal mid-LV short-axis views at the level of the midventricle in (Left) systole and (Right) diastole shows to-and-fro flow between the LV and RV dissection cavities. LV = left ventricular; RV = right ventricular; TTE = transthoracic echocardiography.

**Figure 5:**
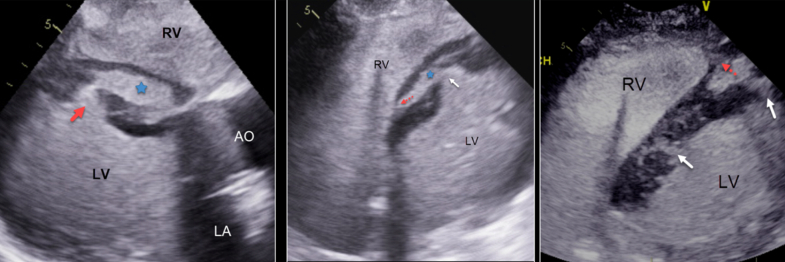
Contrast-Enhanced Images of the VSD and Dissections (Left) Parasternal long-axis view shows VSD inlet (red arrow) that leads to midseptal dissection cavity (blue star). (Middle) Off-axis 4-chamber view shows VSD opening (white arrow) into interventricular septum with dissection cavity (blue star) and exit toward RV base (red arrow). (Right) Off-axis 4-chamber view shows the VSD midseptal and apical entries (white arrows) and exit into the apical RV (red arrow). Contrast reveals a network of channels that dissect and infiltrate the infarcted myocardium. AO = aorta; LA = left atrium; LV = left ventricle; RV = right ventricle; VSD = ventricular septal defect.

Postintervention TTE was performed for further characterization of patient's cardiogenic shock and for identification of suspected intracardiac shunt given right heart catheterization findings. TTE demonstrated hyperdynamic segmental LV function except for the mid to apical septum, which showed akinesis, rupture, and mid wall dissection. A large VSD originated at the mid anteroseptum, and a smaller defect was also identified apically (Figure 3, Video 1). The midseptal dissection drained into a dissection cavity that extended anteriorly to the RV free wall (Videos 2 and 3). To-and-fro flow was detected at the origin of the RV dissection cavity (Figure 4, Video 3). Echocardiography with contrast agent (Definity, Lantheus Medical Imaging) demonstrated the origin and course of the VSD, which included a large dissection cavity within the ventricular septum and the network of channels through the midseptum (Figure 5, Video 4). Contrast within the LV cavity appeared to directly penetrate the infarct tissue. A second, smaller VSD was detected apically; it entered a second septal dissection cavity before draining into the apical RV (Figure 5, Video 4).

**Figure 6:**
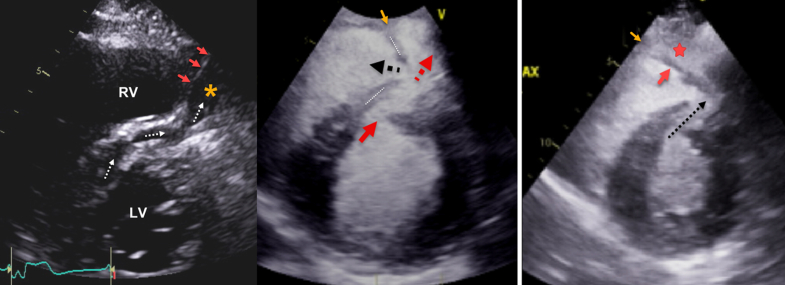
Parasternal Short-Axis Views (Left) Image without contrast shows VSD and septal dissection (dotted white arrows) that continues into an anterior RV dissection cavity (orange star) and begins at the anterior RV septal insertion point (red asterisk). The RV dissection cavity is demarcated from the RV cavity (red arrows). (Middle) Image with contrast shows the course of the VSD connection (red arrow) to the RV dissection cavity (dotted red arrow) and the outlet into the RV cavity (black arrow). The RV dissection cavity is demarcated anteriorly (orange arrow) from the RV. (Right) Image with contrast agent shows more extensively the flap of tissue (red arrow) that separates the RV from the RV dissection cavity (red star). The VSD and the septal dissection are also shown (black arrow). The RV dissection cavity is demarcated anteriorly (orange arrow) from the RV. RV = right ventricle; VSD = ventricular septal defect.

**Figure 7:**
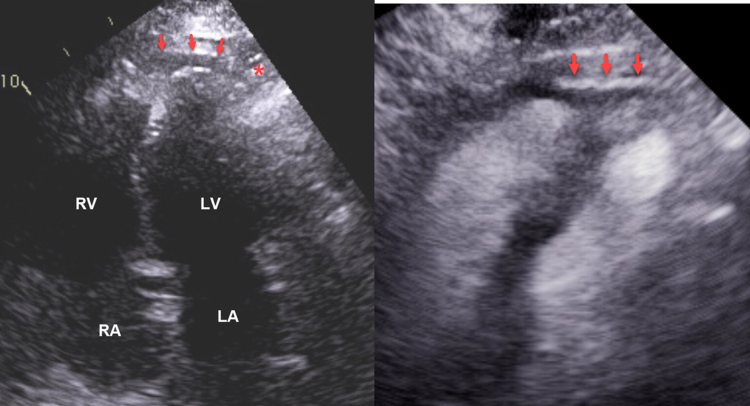
Apical 4-Chamber Views Without (Left) and With (Right) Contrast The images show a thin dissection cavity at the apex (red arrows) that extends to the lateral LV apical epicardium (red asterisk) Contrast and surrounding myocardial contraction (Video 5) indicates that this cavity is not localized effusion or fat. LA = left atrium; LV = left ventricle; RA = right atrium; RV = right ventricle.

Contrast images enhanced the definition of the LV and RV dissections. At the level of the midseptum, contrast delineated the boundaries of the RV dissection and its extension to the anterior RV free wall (Figure 6, Video 3). Separately, a thin layer of contrast also showed dissection that involved the apical RV and LV free walls and demonstrated adjacent myocardial contraction (Figure 7, Video 5). Other findings included severe RV dysfunction, no valvular regurgitation, and no pericardial effusion (Video 6).

## Management

After review of TTE findings, a multidisciplinary heart team determined that the patient was a poor candidate for surgery, with future consideration of percutaneous closure if the patient stabilized. Computed tomography or magnetic resonance imaging may have been beneficial for further characterization of the defect, however they were not performed given the patient's clinical instability.

## Outcome and Follow-Up

Despite optimal medical therapy, the patient's condition worsened. He was resuscitated from 3 episodes of asystolic cardiac arrest over 24 hours. After discussion with family, he was transitioned to hospice care and died 3 days after his admission.

## Discussion

Our case report illustrates how echocardiographic contrast can delineate findings of ventricular septal rupture and dissection with extension to the RV. To our knowledge, 2 previous case reports have been published that describe how echo contrast facilitates recognition of ischemic myocardial rupture. In one case,^1^ contrast agent enabled detection of a through-and-through VSD. In the other case,^2^ contrast extrusion into the inferolateral myocardial wall at the site of myocardial rupture was demonstrated.

Prior case reports on RV dissection have emphasized a multimodality approach to diagnose the extent of RV dissection with computed tomography or three-dimensional transesophageal echocardiography. Most of the time, RV dissection is associated with a basal inferior infarct.^3^ Our case originated in the mid and apical septum adjacent to the anterior RV insertion point into the anteroseptum. Our experience indicates that two-dimensional echocardiography with contrast generates high-resolution and detailed images that may complement other approaches.

A spectrum of remodeling changes occurs in the wall of acutely infarcted myocardium. These include increased wall thickness due to myocardial edema and loss of contractile function, and changes in myocardial texture.^4^^,^^5^ We previously reported that these findings may be accompanied by direct contrast penetration into the myocardium and a bandlike separation of the endocardium from midmyocardial layer, consistent with intramyocardial hemorrhage.^6^ Our present case illustrates the other end of the spectrum, with multiple ruptures of the septum, septal canalization and contrast penetration, and RV and LV dissection.

Our patient presented with angiographic evidence of occlusion of a right coronary artery–saphenous vein graft and troponin elevation, as well as electrocardiographic changes consistent with MI. Detection of an oxygen step-up on right heart catheterization suggested a postinfarction intracardiac shunt. TTE confirmed the presence of complex postinfarction VSDs, with evidence of intricate dissection channels, blood pooling within the septum, and the formation of an intramyocardial cavity consistent with RV free wall dissection (Figures 3, 4, 6, Videos 1 and 2). Guidelines recommend that TTE is performed first for MI patients in cardiogenic shock. In our patient, STEMI (ST-segment elevation myocardial infarction) code activation in the middle of the night resulted in his rushed transfer to the catheterization laboratory before echocardiography could be performed.^7^

Surgical repair is the treatment of choice in these cases, although mortality rates remain high.^8^ Transcatheter repair may serve as a bridge to surgery or as a definitive therapy in select patients.^9^ Temporization with extracorporeal membrane oxygenation or LV assist devices may enable fibrosis of the infarcted tissue and the stability of surgical repair or percutaneous insertion of a VSD occluder. Our team determined that the patient was a poor surgical candidate and therefore would not benefit from mechanical support bridging.

Buda et al^10^ wrote a thorough review of the management of these patients and noted that early intervention may not succeed because of the friability of edematous tissue. Percutaneous closure was considered in our patient, but the extent of infarcted tissue and his hemodynamic and electrical instability prompted the decision for palliative care.

## Conclusions

Mechanical complications of MI are rare but often fatal, especially when multiple anatomical defects are present. In our case, two-dimensional contrast-enhanced echocardiographic imaging improved anatomical resolution, facilitated diagnosis, and guided critical decisions regarding intervention. Early and multimodal imaging are indispensable in managing such complex postinfarction presentations.

## Funding Support and Author Disclosures

Dr Cohen has stock ownership in Pfizer, Boston Scientific, Edwards Lifesciences, Medtronic, and Abbott. All other authors have reported that they have no relationships relevant to the contents of this paper to disclose.

## Appendix

Video 1 Parasternal Long-Axis Views Showing Midcavity VSD and Dissection Through the Septum Toward the RV (Top Left) Two-dimensional echocardiography only. (Top Right) Two-dimensional with contrast. (Bottom Left) Three-dimensional echocardiography. (Bottom Right) Two-dimensional with color Doppler.

Video 2 Parasternal Short-Axis Views at the Basal (Left), Mid (Center), and Apical (Right) Levels Showing the Septal Dissection (Left) and Its Extension Into the RV Anterior Wall (Center) The dissection is not evident apically (right). Also refer to Figure 6.

Video 3 Parasternal Short-Axis Views With 2D (Left), Color Doppler (Middle), and Contrast (Right) Showing the Septal Dissection and Demarcating the RV Dissection Cavity and Its Connection With the Main RV Cavity.

Video 4 Contrast-Enhanced Parasternal Long-Axis (Left) and Off-Axis 4-Chamber Views (Middle and Right) Showing the VSD and Septal Dissection The middle video also shows a network of channels that dissect and infiltrate the infarcted myocardium. Also see Figure 5.

Video 5 Apical 4-Chamber View Without (Left) and With (Right) Contrast Showing a Thin Dissection Cavity at the Apex Contrast and surrounding myocardial contraction indicate that this cavity is not localized effusion or fat. Also see Figure 7.

Video 6 Case Presentation Including a Composite of Videos.
